# Immunomodulatory Effect of Proteasome Inhibitors via the Induction of Immunogenic Cell Death in Myeloma Cells

**DOI:** 10.3390/ph16101367

**Published:** 2023-09-27

**Authors:** Maiko Matsushita, Sho Kashiwazaki, Satoshi Kamiko, Michio Kobori, Makoto Osada, Hisako Kunieda, Maki Hirao, Daiju Ichikawa, Yutaka Hattori

**Affiliations:** 1 Division of Clinical Physiology and Therapeutics, Faculty of Pharmacy, Keio University, Tokyo 105-8512, Japan; 2 Department of Hematology, Tokyo Saiseikai Central Hospital, Tokyo 108-0073, Japan; makoosd.0221@gmail.com (M.O.);; 3 Department of Health Science, Faculty of Sports and Health Science, Daito Bunka University, Saitama 355-8501, Japan

**Keywords:** immunogenic cell death, myeloma, proteasome inhibitors, immunomodulatory, carfilzomib, bortezomib

## Abstract

Several anti-cancer drugs are known to have immunomodulatory effects, including immunogenic cell death (ICD) of cancer cells. ICD is a form of apoptosis which is caused by the release of damage-associated molecular patterns (DAMPs), the uptake of cancer antigens by dendritic cells, and the activation of acquired immunity against cancer cells. ICD was originally reported in solid tumors, and there have been few reports on ICD in multiple myeloma (MM). Here, we showed that proteasome inhibitors, including carfilzomib, induce ICD in myeloma cells via an unfolded protein response pathway distinct from that in solid tumors. Additionally, we demonstrated the potential impact of ICD on the survival of patients with myeloma. ICD induced by proteasome inhibitors is expected to improve the prognosis of MM patients not only by its cytotoxic effects, but also by building strong immune memory response against MM cells in combination with other therapies, such as chimeric antigen receptor—T cell therapy.

## 1. Introduction

The newly developed drugs such as immunomodulatory drugs (IMIDs), proteasome inhibitors (PIs), histone deacetylase inhibitors, and monoclonal antibody-based drugs have considerably improved the survival rates of patients with multiple myeloma (MM) [1]. Additionally, T cell-mediated therapies, such as stem cell transplantation and chimeric antigen receptor (CAR)-T cell or bispecific T cell engager (BiTE) therapy, have shown clinical potential, suggesting that myeloma cells are highly immunogenic [2,3,4]. However, myeloma is still an incurable disease, and most patients suffer from disease relapse after standard therapies [5]. Therefore, effective treatments are needed to improve the survival of patients with myeloma. Here, we focused on the immunological phenomenon known as immunogenic cell death (ICD). Certain chemotherapeutic drugs, such as anthracyclines, do not only cause cell death, but also facilitate cancer cells to increase calreticulin (CRT) expression on the cell surface and release of damage-associated molecular patterns (DAMPs). DAMPs include high mobility group box 1 (HMGB1) and adenosine triphosphate (ATP). Most DAMPs originally exist inside the cells. They are released to outside of the tumor cells during the course of apoptosis. DAMPs can bind to pattern recognition receptors (PRR) on antigen presenting cells and facilitate maturation or activation of immune cells. In ICD setting, CRT and HMGB1 act as “eat me” signals, and ATP works as a “find me” signal that leads the recognition of cancer cells by antigen-presenting cells [6,7]. Finally, activated T cells kills cancer cells and immune memories against cancer remains. This phenomenon has primarily been reported in several types of solid cancers including breast cancer or small-cell lung cancer [8,9,10,11]. Especially, endoplasmic reticulum (ER) stress is known to trigger CRT expression in these malignancies [12,13]. Evoking ICD by drugs would strengthen the effectiveness of cancer treatment. However, there is limited evidence on ICD in haematological malignancies such as MM [14,15,16]. In this study, we aimed to ascertain which anti-myeloma drugs most effectively induce ICD. We also tried to delineate the mechanism underlying the induction of immunological responses by these drugs in myeloma cells. Accordingly, we evaluated a panel of anti-myeloma drugs for their ability to induce ICD in myeloma cells using human myeloma cells and immune cells, and outline the plausible cellular signaling induced by PIs. Furthermore, we analyzed the effects of ER stress-related genes on prognosis of myeloma patients treated with PIs. These analyses may provide a strong rationale for using ICD inducers for the treatment of myeloma patients.

## 2. Results

### 2.1. Treatment with Proteasome Inhibitors Most Induced ICD in Myeloma Cells

**Figure 1:**
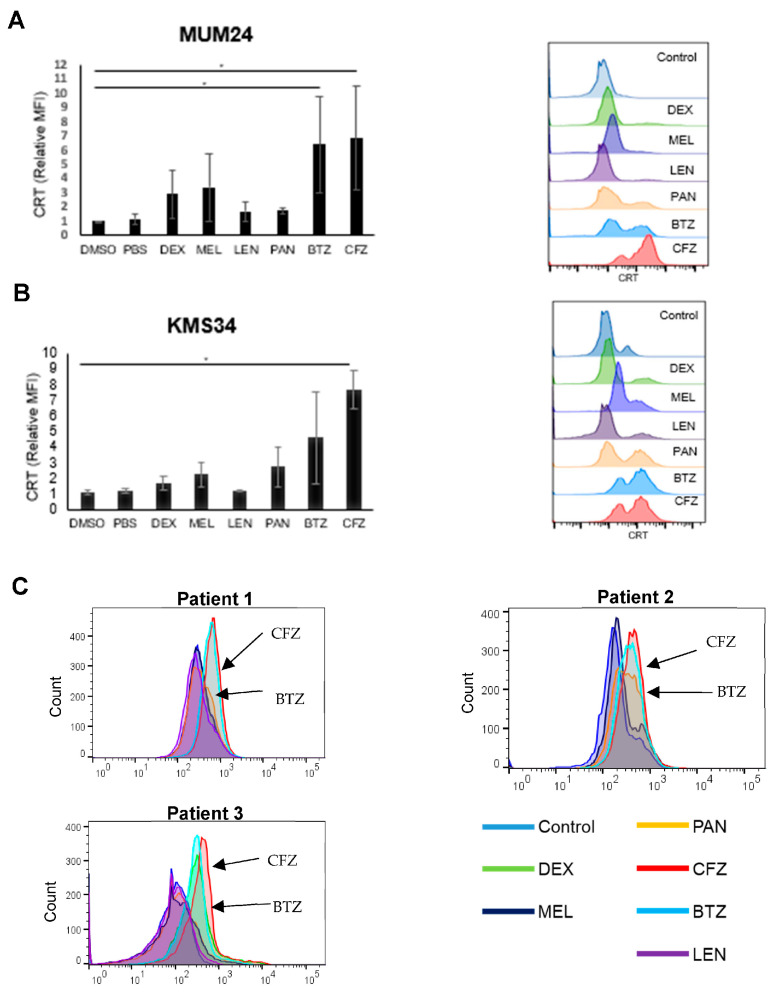
Proteasome inhibitors induce cell surface expression of calreticulin in myeloma cells. (**A**,**B**) MUM24 and KMS34 were treated with anti-myeloma drugs (dexamethasone;DEX, melphalan;MEL, lenalidomide;LEN, panobinostat;PAN, bortezomib;BTZ, and carfilzomib;CFZ) at an IC50 of each drug for 48 h. KMS34 was not treated with LEN, since it is LEN-resistant. CRT expression detected using flow cytometry (right panel) and mean fluorescence intensity (MFI) relative to that of control (PBS for DEX, DMSO for MEL, LEN, PAN, BTZ, and CFZ) is indicated (left panel). Data represent the mean and standard error of mean (s.e.m.) of three independent experiments. * *p*-value < 0.05. (**C**) CD138^+^ cells were purified from patients’ bone marrow samples and treated with anti-myeloma drugs for 48 h. Samples were stained with anti-CRT monoclonal antibody and analyzed using flowcytometry.

In order to compare the ability of anti-myeloma drugs to induce ICD, we first detected the cell surface expression of CRT, which is commonly used as an indicator of ICD [8]. Human myeloma cell lines MUM24 and KMS34 were treated with each of anti-myeloma drugs: dexamethasone, melphalan, lenalidomide, panobinostat, bortezomib, and carfilzomib at IC50. We found that PIs (bortezomib and carfilzomib) were the strongest inducers of CRT in both cell lines (Figure 1A,B).

We confirmed that these drugs could induce ICD in CD138^+^ cells obtained from the bone marrow samples of patients. The samples were collected during diagnosis or relapse, and all patients responded to treatment regimen including bortezomib. For all three cases examined, CRT expression on the surface of myeloma cells increased markedly after treatment with 3 nM (bortezomib) or 4 nM (carfilzomib) of the PIs (Figure 1C).

**Figure 2:**
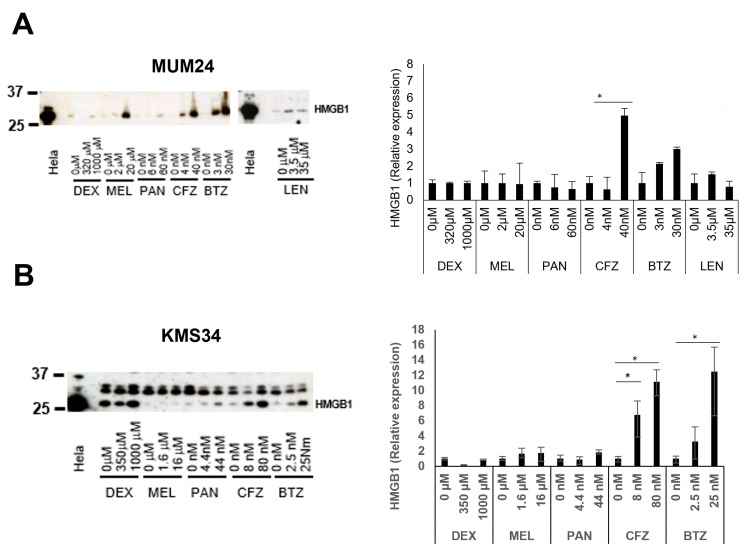
Proteasome inhibitors induce secretion of HMGB1 from myeloma cells. (**A**,**B**) Cell culture medium collected after treatment with anti-myeloma drugs for 24 h and HMGB1 protein detected using western blot (left panel). Protein extracted from Hela cells was used as a loading control. LEN was not used for KMS34 since this cell line is resistant to LEN. Relative expression level compared with control (PBS for DEX, and DMSO for other drugs) is indicated (right panel). Data represent the mean and standard error of mean (s.e.m.) of three independent experiments. * *p*-value < 0.05.

Thereafter, we examined the secretion of drug-induced HMGB1, which is another well-known indicator of ICD. Lenalidomide was not used in KMS34, since this cell line is resistant to the drug. ln both cell lines, HMGB1 was highly released after treatment with bortezomib or carfilzomib (Figure 2). In particular, carfilzomib could induce secretion of HMGB1 from KMS34 cells even with a low dose (5 nM).

### 2.2. Myeloma Cells Treated with Anti-Myeloma Drugs Facilitated Activation of DC

**Figure 3:**
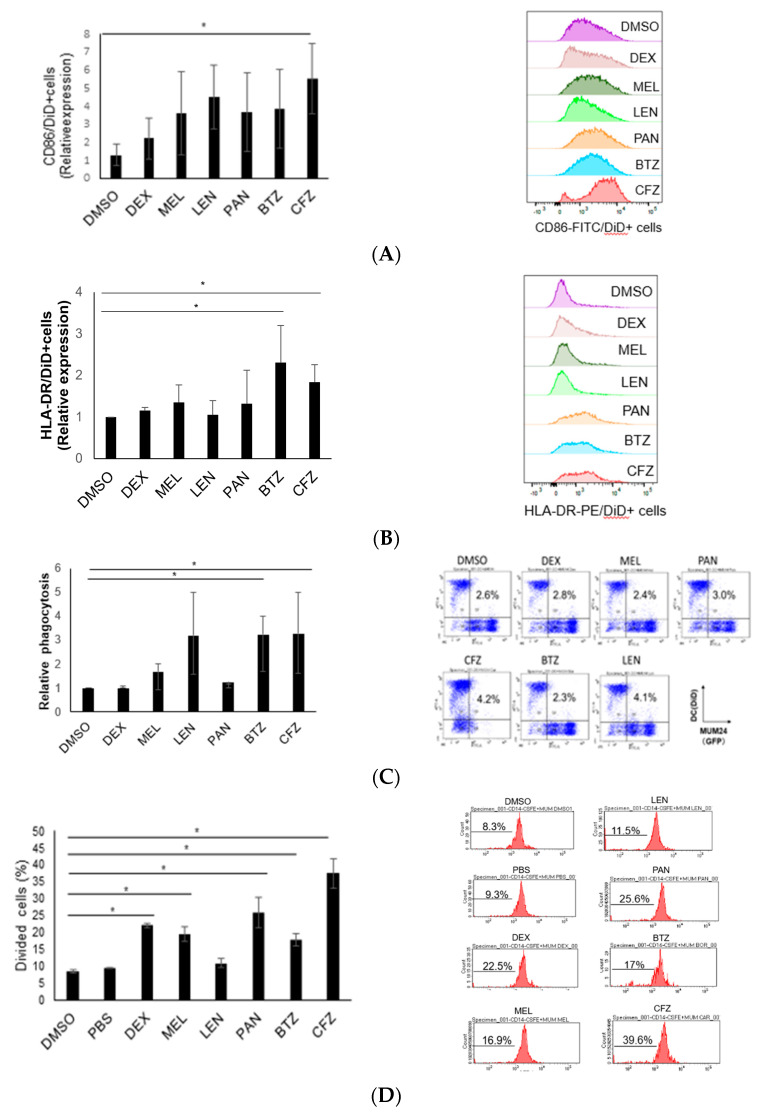
Treatment of myeloma cells with anti-myeloma drugs enhanced antigen presentation by dendritic cells and T cell division. (**A**,**B**) Monocyte−derived dendritic cells (DCs) from healthy donors were labeled with DiD and co-cultured with MUM24 treated with each drug for 48 h and stained with FITC-conjugated anti−CD86 monoclonal antibody or PE-conjugated anti−HLA−DR monoclonal antibody, then analyzed by flow cytometry. DiD^+^ cells were gated and CD86 or HLA−DR expression on DiD^+^ cells were evaluated and mean fluorescence intensity (MFI) relative to that of isotype control antibody are indicated (left). Representative raw data of histogram is also shown (right). (**C**) Monocyte−derived DCs were labeled with DiD and co−cultured with GFP−expressing MUM24 treated with each drug for 48 h and percentage of DiD^+^ GFP^+^ cells are shown as indicator of phagocytosis. Relative data compared with control (PBS for DEX, and DMSO for other drugs) is indicated (left). Representative data of dotplpt is also shown (right). (**D**) CFSE−labeled CD14^−^ cells were co−cultured with CD14^+^ cells−derived DCs pulsed with MUM24 which were treated with each drug at IC50 for 5 days and analyzed by flow cytometry. Percentage of CFSE-low cells in CD3^+^CD8^+^ cells is indicated (left). Representative data of histogram is also shown (right). Data represent the mean and standard error of mean (s.e.m.) of three independent experiments. * *p* < 0.05.

Recently, several reports have shown that CRT expression is induced in myeloma cells [14,16]. However, there is little evidence on actual immune activation by ICD of myeloma cells. Therefore, we examined whether the induction of CRT expression on myeloma cells could activate DCs and T cells. Monocyte-derived DCs from a healthy donor were labelled with DiD and co-cultured with MUM24 cells that are pre-treated with anti-myeloma drugs. DCs cocultured with carfilzomib-treated MUM24 cells showed higher expression of CD86 and HLA-DR (Figure 3A,B). In order to evaluate phagocytosis by DCs, we next added green fluorescent protein (GFP)-expressing MUM24 cells, pre-treated with anti-myeloma drugs, to DiD—labeled DCs. We observed that DCs showed a higher phagocytic activity against MUM24 cells treated with bortezomib or carfilzomib (Figure 3C). Lenalidomide-treated MUM24 cells were also engulfed by DCs; however, the difference was not significant.

We next evaluated whether T cells could expand when they are co-cultured with DCs pulsed with myeloma cells treated with anti-myeloma drugs. The CFSE−labelled CD8^+^ cells from healthy donors divided when they were co-cultured with DCs pulsed with MUM24 cells treated with dexamethasone, melphalan, panobinostat, bortezomib and carfilzomib. Among the drugs, carfilzomib most strongly stimulated T cell division (Figure 3D).

### 2.3. Specific Pathway of Unfolded Protein Response (UPR) Was Related to ICD Induction in Myeloma Cells

**Figure 4:**
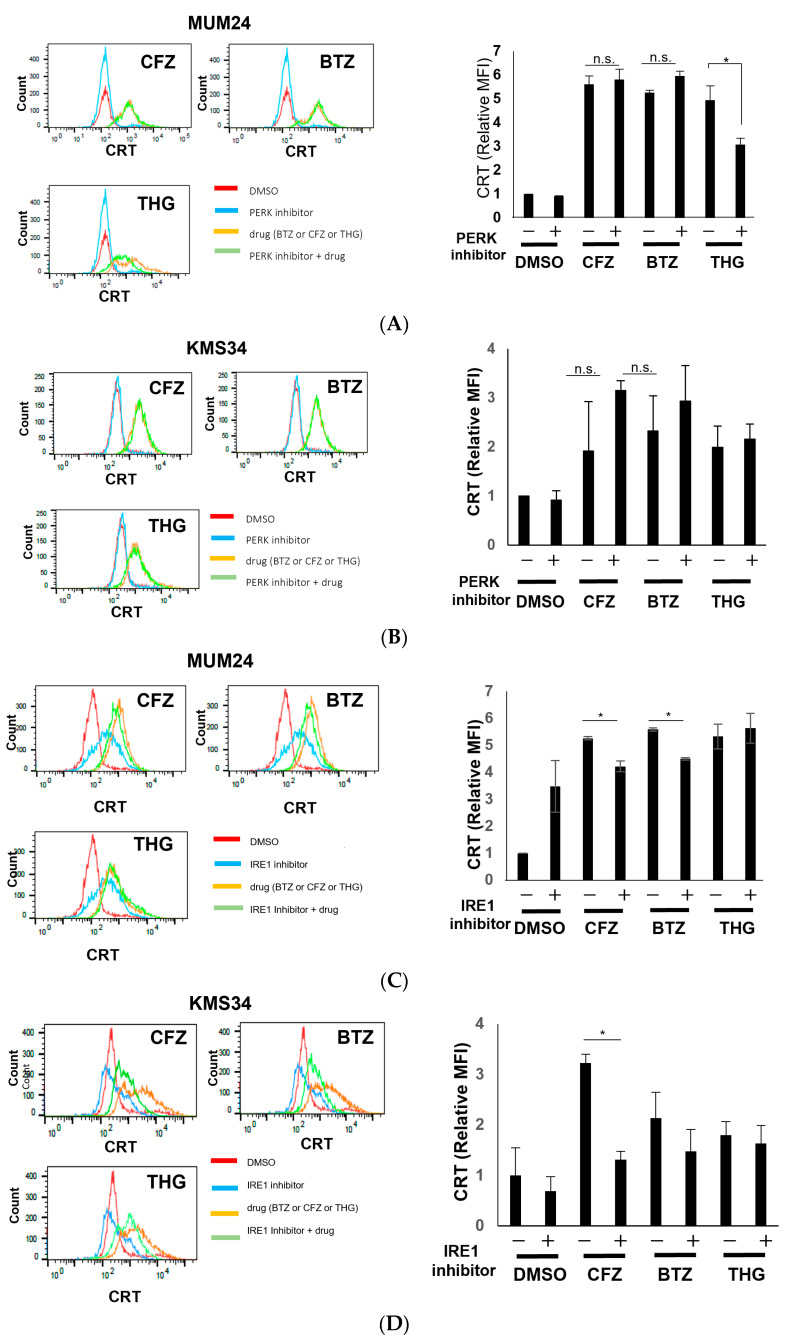
IRE1-XBP1 pathway of unfolded protein responses (UPRs) induced by PIs affects ICD in myeloma cells. MUM24 (**A**,**C**) or KMS34 (**B**,**D**) were treated with PERK inhibitor (GSK2606414) or IRE1α inhibitor (STF083010) one hour before addition of proteasome inhibitors (BTZ, CFZ) and CRT expression was detected after 48 h by flow cytometry (left). MFI relative to that of DMSO is indicated (right panel). Thapsigargin (THG) was used as a positive control of ER stress inducer. Data represent the mean and standard error of mean (s.e.m.) of three independent experiments. * *p*-value < 0.05, n.s. = not significant.

To clarify the mechanism underlying the strong ICD-inducing effects of PIs in myeloma, we investigated the signaling pathways related to endoplasmic reticulum (ER) stress [17]. Since drugs other than PIs did not induce strong CRT expression or HMGB1 secretion, we focused on bortezomib and carfilzomib. Protein kinase RNA-like endoplasmic reticulum kinase (PERK)-eukaryotic initiation factor 2α (eIF2α) pathway of the unfolded protein response (UPR) has been reported to be essential in CRT expression of solid tumors [18,19,20]. Therefore, we assessed the effect of the PERK inhibitor GSK2606414 on PI-dependent CRT expression. In both MUM24 and KMS34, the expression level of CRT was not downregulated by pre-treatment with the PERK inhibitor, GSK2606414 (Figure 4A,B). Contrastingly, CRT expression induced by thapsigargin (THG), a strong inducer of ER stress and UPR, was inhibited by addition of GSK2606414 in MUM24. We next evaluated the inositol-requiring enzyme 1-α (IRE1α)-X-box protein 1 (XBP1) pathway, which is another UPR pathway. In response to ER stress, the RNase domain of IRE1α is activated and 26 nucleotides in XBP1 mRNA are deleted, thereby producing a spliced form of XBP1 (XBP1s) [21]. IRE1α inhibitor, STF083010, is known to suppress splicing or XBP1. We observed that CRT expression on the surface of MUM24 cells and KMS34 cells, which was induced by carfilzomib or bortezomib, was significantly suppressed by the addition of STF083010, IRE1 inhibitor (Figure 4C,D).

### 2.4. Gene-Expression Profile of Myeloma Cells Treated with Proteasome Inhibitors

**Figure 5:**
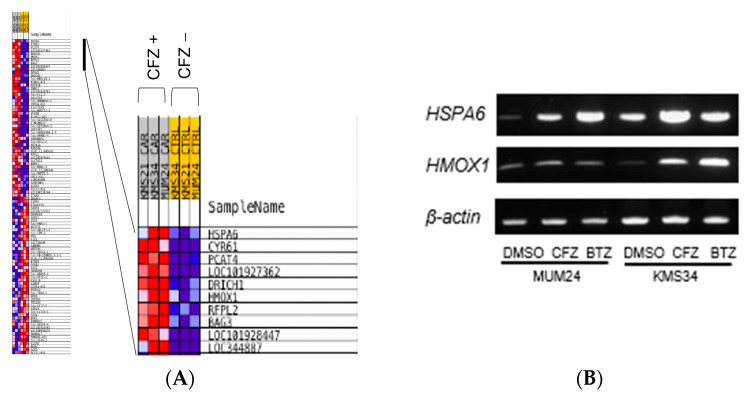
UPR-related genes are upregulated in myeloma cells after treatment with Pis. (**A**) Clustering of differential genes from DNA microarray analysis was analyzed (Agilent Array, SurePrint G3 Human Gene Expression 8 × 60K v3). Left panel is the heatmap of all significantly upregulated or downregulated genes after treatment of MUM24, KMS34, and KMS21 with carfilzomib for 6 h. Right panel shows a part of upregulated genes containing ER stress-related genes (*HSPA6*, *HMOX1*). (**B**) The mRNA expression level of *HSPA6* and *HMOX-1* was confirmed by RT-PCR. *β-actin* was an internal control.

Since it was suggested that ICD was induced after treatment with PIs in myeloma cells via IRE1-XBP1 pathway, which is different from the observation in solid tumors, DNA microarray analysis was performed to further clarify the genes induced in ICD of myeloma cells. We found that carfilzomib upregulated the expression of several genes related to UPR, including *HSPA6* and *HMOX1*, in myeloma cell lines (Figure 5A). RT-PCR analysis confirmed that the expression of these genes increased after treatment with carfilzomib or bortezomib (Figure 5B).

### 2.5. Expression of UPR-Related Genes were Correlated Positively with Overall Survival of Myeloma Patients

**Figure 6:**
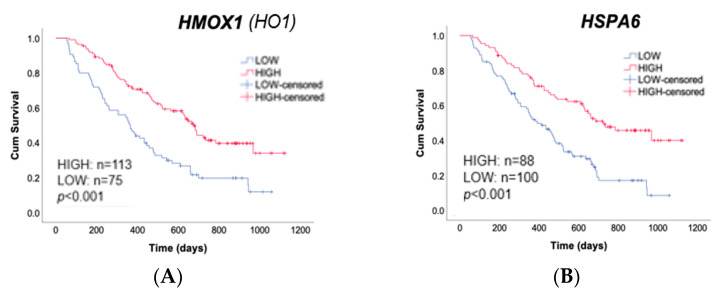
UPR-related genes are upregulated in myeloma cells after treatment with Pis. Gene expression data and clinical data of myeloma patients treated with bortezomib (GSE9782) was analyzed using SPSS ver.4 software. The patients were divided into high (red line) or low (blue line) quantile expressions of *HMOX1* gene (**A**) or *HSPA6* gene (**B**). Overall survival rate was compared using Kaplan–Meier survival plots.

The relationship between the expression levels of *HSPA6* or *HMOX1* genes and prognosis of myeloma patients treated with proteasome inhibitors was assessed using gene expression data and clinical information obtained from the public database (GSE9782) [22]. High expression of both *HMOX1* and *HSPA6* correlated positively with overall survival of myeloma patients (Figure 6A,B).

## 3. Discussion

Despite the development of novel immune therapies, such as IMiDs, PIs, or monoclonal antibodies, and CAR-T therapies, a certain part of myeloma patients have poor prognosis [1]. Therefore, identification of the drugs which could facilitate the effect of these treatment is necessary. Some kinds of chemotherapeutic drugs, such as anthracyclines, have been reported to activate immune responses via ICD of cancer cells in solid tumors [8]. However, little is known in hematological malignancies. Gulla et al. recently reported that bortezomib induced ICD in myeloma cells [16]. However, there are no reports which compare all of the main anti-myeloma drugs including carfilzomib side by side with regard to the ability of inducing ICD. 

Our results indicate that a relatively low concentration of bortezomib and carfilzomib can enhance expression of CRT on cell surface of myeloma cell lines and some patients’ samples. Myeloma cells treated with these PIs also secreted a high level of HMGB1. Since there are other ICD markers, including type-I IFN, CXCL10, IL-1β, and IL-17, further study to detect these markers might strengthen our findings in the future.

Importantly, these drugs could induce ICD in KMS34 cells at an IC50 (carfilzomib: 8 nM, bortezomib: 2.5 nM) that was much lower than the maximum concentration used in clinical settings (carfilzomib: 1–4 μM, bortezomib: 0.05–0.5 μM). The cell viability was almost the same after treatment with each drug since we used a dose of IC50 which we had decided for each myeloma cell line. However, the amount of CRT and HMGB1 was highest in PI-treated myeloma cells. On the other hand, myeloma cells treated with dexamethasone showed little amount of CRT expression and HMGB1 secretion, despite that the cell viability was same as those treated with other drugs. This discrepancy has also been observed in cisplatin-treated lung cancer cells [23]. The disparities among anti-cancer drugs might be derived from the different involvement of ER stress and UPR in cell death triggered by each drug. 

Taken together, PIs are strong ICD inducers compared to other anti-myeloma drugs, despite that all the drugs exert a cytotoxic effect against myeloma cells. 

**Figure 7:**
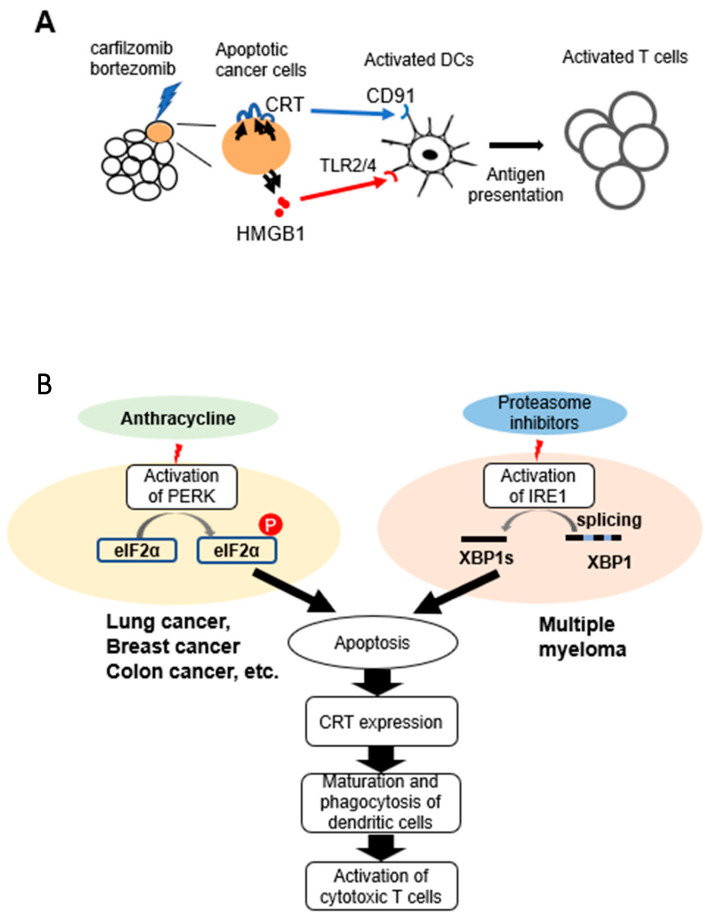
Possible mechanism underlying ICD induction in myeloma cells by Pis. (**A**) Carfilzomib or bortezomib could trigger expression of CRT or secretion of HMGB1 in myeloma cells and induce activation of DCs, leading to T cell responses against myeloma cells. (**B**) PIs activate IRE1a and subsequent splicing of XBP1, followed by CRT expression, which is distinct from solid tumors, in which phosphorylation of eIF2α plays an important role for CRT expression.

CRT and HMGB1 are known to bind to receptors, CD91 and Toll-like receptor 2/4 on DCs and facilitate maturation and activation of DCs (Figure 7A). There have been no reports which compared the effects of anti-myeloma drugs including both carfilzomib and bortezomib on maturation of human dendritic cells. Therefore, we co-cultured human monocyte-derived DCs with myeloma cells treated with each drug. Although we observed some variety in altitude of immune response in each experiment because of using human blood samples, we observed statistically significant differences in the assays with bortezomib or carfilzomib. The expression of the maturation marker, CD86 and HLA-DR, in DCs was upregulated after coculture with PI-treated MUM24 cells. Interestingly, carfilzomib tends to induce stronger maturation of DCs. It might be caused by irreversible blockade of proteasome of carfilzomib [24]. We also found that MUM24 cells treated with bortezomib and carfilzomib were effectively engulfed by DCs, which coincides with upregulation of maturation markers in those DCs. Moreover, T-cells divided when they were co-cultured with DCs pulsed with carfilzomib-treated myeloma cells. This phenomenon was not observed when myeloma cells were treated with lenalidomide, although DCs engulfed lenalidomide-treated myeloma cells. There is a possibility that LEN remaining in supernatant of drug-treated myeloma cells enhanced expression of CD86 and phagocytosis of DC at some content; however, lenalidomide did not induce enough DC activation because the amount of HMGB1 secreted from myeloma cells after lenalidomide treatment was less than those by PIs in MUM24 (Figure 2A). We also confirmed that each drug except melphalan did not directly affect proliferation of human T cells, Jurkat cell line (Supplementary Figure S1A).

Furthermore, we investigated the signaling pathways induced in myeloma cells by bortezomib and carfilzomib. PIs are known to induce ER stress [17], and subsequently UPR occurs, that plays an important role in CRT expression [25]. In particular, PERK- eIF2α pathway has been reported as a key pathway in ICD induction of solid tumors [8]. For example, it has been shown that blocking PERK activity via GSK2606414 abolishes oleandrin-dependent CRT expression in breast cancer cells [19]. Additionally, Panaretakis et al. reported that inhibiting PERK activity with shRNA downregulates CRT expression in colon cancer cells after mitoxantrone treatment [26]. In our study, there was no change in the level of CRT expression by PERK inhibitor in MUM24 or KMS34 despite that a reduction in the phosphorylation of eIF2α was observed (Supplementary Figure S2A,C). Contrastingly, CRT expression induced by THG, a strong inducer of ER stress and UPR, was inhibited by addition of the PERK inhibitor in MUM24. Therefore, we next evaluated IRE1α-XBP1 signaling pathway, which is upregulated in myeloma cells [27]. We observed that CRT expression on the surface of MUM24 and KMS34, as induced by PIs, was partially suppressed by the addition of IRE1α inhibitor, STF083010, which suppressed splicing of XBP1 in MUM24 and KMS34 (Supplementary Figure S2B,D). Although there might be another pathway affecting CRT expression of myeloma cells, these results suggest that the IRE1α-XBP1 pathway may play a more important role in the ICD of myeloma cells than the PERK-eIF2α pathway, indicating that PIs may induce CRT expression in myeloma cells by the different UPR pathway from other types of cancer cells (Figure 7B). Since myeloma cells are subjected to greater ER stress than solid tumors due to the excessive M-protein production [25], the UPR characteristics might not be the same as those in other cancers. It has also been suggested that upregulation of IRE1α-XBP1 pathway is related to immune modulation via NF-κB signaling [28], which supports the immunostimulatory effects of PIs. Recently, the complex of disulfiram and copper (DS/Cu) is also reported to induce apoptosis of myeloma cells by inhibiting proteasome [29,30]. In particular, copper (II) ions suppress peptidase activities of 20 S proteasomes [31]. On the other hand, Matos et al. reported that copper sulfate could enhance splicing of XBP1 in WI-38 fibroblast cells [32]. Therefore, it is of interest to evaluate whether DS/Cu could induce ICD in myeloma cells via IRE1α-XBP1 pathway.

Thereafter, DNA microarray analysis was performed to further clarify the genes induced in ICD by PIs. We found that carfilzomib upregulated the expression of several genes related to UPR, such as *HSPA6* (heat shock protein family A (Hsp70) member 6) and *HMOX1* (hemo oxygenase 1), in myeloma cell lines. Interestingly, these genes were not included in the gene set induced by bortezomib which was previously reported [16], suggesting that carfilzomib could evoke ICD via different pathway as bortezomib. In fact, our data showed that carfilzomib induced higher amount of cell surface expression of CRT and secretion of HMGB1 in MUM24 cells. 

Then, we evaluated the relationship between the expression levels of these genes and prognosis of myeloma patients using gene expression data and clinical information obtained from the public database (GSE9782). High expression of *HMOX1* or *HSPA6* was correlated positively with overall survival. Notably, *HMOX1* encodes a chaperone protein which controls folding of proteins, and the gene is upregulated by ER stress as a downstream target of the IRE1α-XBP1 pathway [33]. HSPA6 also plays a role in assembly of proteins in ER-associated protein degradation (ERAD) process, which occurs during UPR.

Our data and the results from these forementioned study suggest that activation of the IRE1α-XBP1 pathway via PIs might contribute to a better clinical course via not only the direct cytotoxicity of those drugs but also through the long-term effects of the immunological memory obtained after ICD of myeloma cells. 

Finally, we suggest the following treatment strategies: the combination of PIs with other immunological treatments, such as monoclonal antibodies, CAR-T therapy, and immune checkpoint inhibitors (ICIs). CAR-T therapy targeting B cell maturation antigen (BCMA) is effective as a treatment method of relapsed or refractory myeloma patients [34]. However, resistance against CAR-T therapy via loss of target antigen or suppression of CAR-T cells by immune-suppressive tumor microenvironment have been suggested [35]. It has also been reported that ICIs could not prolong the survival of myeloma patients in clinical trials even with lenalidomide [36]. Therefore, novel strategies are needed to overcome these problems. Srivastava et al. have shown that oxaliplatin, known as a strong ICD inducer, could improve the efficacy of receptor tyrosine kinase-like orphan receptor 1 (ROR1)-specific CAR-T therapy in combination with ICIs in breast and lung cancer in mice model [37]. PIs, as ICD inducers, might be also useful in treatment with BCMA-specific CAR-T therapy or ICIs for myeloma patients. It should be clarified in future clinical trials.

In addition, our gene expression analysis revealed that the genes related to IRE1α-XBP1 pathway are related to prognosis of myeloma patients. The detection of UPR-related genes, including *HMOX1*, may be used as a biomarker for predicting clinical benefit in this setting.

In conclusion, PIs can promote immune reactions in myeloma cells by inducing ICD. Moreover, we showed the possible correlation between XBP1-IRE1α signaling and ICD in myeloma cells for the first time. Our findings provide a strong rationale for using PIs with other immunotherapies to improve the survival of patients with myeloma.

## 4. Materials and Methods

### 4.1. Cell Lines

MUM24 was established in our laboratory from a thalidomide-resistant myeloma patient [38]. KMS34 were provided by Dr. Ohtsuki of Kawasaki Medical School (Kurashiki, Japan). Human T cell line, Jurkat, was purchased from American Type Culture Collection. These cell lines were maintained at 37 °C in a humidified atmosphere of 5% CO_2_ in RPMI-1640 medium (Sigma-Aldrich, Saint Louis, MO, USA) and 10% foetal bovine serum (FBS) (Invitrogen™, Life Technologies, Grand Island, NY, USA).

### 4.2. Reagents

Melphalan (Sigma-Aldrich), lenalidomide (Santa Cruz Biotechnology, Dallas, TX, USA), panobinostat (Selleck Chemicals, Houston, TX, USA), bortezomib (Selleck Chemicals), and carfilzomib (Amgen, Thousand Oaks, CA, USA) were dissolved in dimethyl sulfoxide (DMSO) (Sigma-Aldrich) and kept frozen at −20 °C. Dexamethasone (MSD, Tokyo, Japan) was stored at 4 °C until use. Thapsigargin (Wako, Osaka, Japan), PERK inhibitor (GSK2606414; Selleck Chemicals) and IRE1α inhibitor (STF083010; Sigma-Aldrich) were also dissolved with DMSO and stored at −20 °C.

### 4.3. Patient Samples

Bone marrow samples were obtained from 3 MM patients treated at Tokyo Saiseikai Central Hospital. Mononuclear cells were isolated from the bone marrow aspirates by centrifugation with Lymphoprep™ (Axis-Shield, Oslo, Norway). CD138^+^ cells were purified by MACS beads and Columns (Miltenyi Biotec, Bergisch Gladbach, Germany). Written informed consent was obtained from all patients. The use of these clinical samples was approved by ethical committee of Tokyo Saiseikai Central Hospital (No. 28-66) and the Faculty of Pharmacy, Keio University (No. 190605-3).

### 4.4. Detection of CRT Expression by Flow Cytometry

MUM24 or KMS34 was treated with anti-myeloma drugs (dexamethasone; DEX, melphalan; MEL, lenalidomide; LEN, panobinostat; PAN, bortezomib; BTZ, and carfilzomib;CFZ) at an inhibitory concentration of 50 (IC50) of each drug for 48 h. Amount of each drug was as follows; MUM24: DEX; 320 μM, MEL; 2 μM, LEN; 3.5 μM, PAN; 6 nM, BTZ; 3 nM, CFZ; 4 nM. KMS34: DEX; 350 μM, MEL; 1.6 μM, LEN; 3.5 μM, PAN; 4.4 nM, BTZ; 2.5 nM, CFZ; 8 nM. KMS34 was not treated with LEN, since it is LEN-resistant. After 48 h, cells were stained with DyLight^TM^ 488-labeled anti-CRT antibody (clone FMC75; Enzo Life Sciences, New York, USA). We gated FSC^low^ SSC^low^ population to get rid of terminally dead cells. CRT expression was detected using BD FACS Celesta™ (Becton Dickinson and Company, Franklin Lakes, NJ, USA) and mean fluorescence intensity (MFI) was measured using FlowJo™ (Version 10.7.1, Becton Dickinson and Company). CD138^+^ cells purified from patients’ bone marrow samples were also used in the same setting as MUM24 and KMS34.

### 4.5. Detection of HMGB1 by Western Blot

HGMB1 secreted by drug-treated myeloma cells was detected by Western blot. A total of 0.8 million per well of myeloma cells were seeded in 12-well plates in RPMI-1640 medium (Sigma-Aldrich) plus 10% FBS. Cell culture medium were collected after treatment with anti-myeloma drugs for 24 h and concentrated by adding acetone (WAKO) for 2 h at −20 °C, centrifuged at 12,000× *g*, and dissolved in 1% NP-40 lysis buffer with protease inhibitor cocktail tablet (F.Hoffman-La Roche Ltd., Basel, Switzerland). After measurement concentrations of protein by BCA protein assay kit (Thermo Fisher Scientific K·K), the protein amount was adjusted by adding 1% NP-40 lysis buffer and then they were mixed with Laemmli buffer (1.35% SDS, 10% glycerol, 2% 2-mercaptoethanol, 0.002% bromophenol blue, 83 mM Tris, pH 6.8). Those samples were boiled for 5 min and separated by sodium dodecyl sulfate-polyacrylamide gel electrophoresis (SDS-PAGE), then transferred to a nitrocellulose membrane (Merck Millipore, Dublin, Ireland). The membrane was blocked using Tris-buffered saline-Tween 20 (TBS-T) containing 5% skimmed milk and treated overnight with anti-HMGB1 antibody (ab18256; Abcam, Cambridge, UK), followed by addition of anti-mouse immunoglobulins/HRP antibody (DAKO, Glostrup, Denmark). ECL Prime Western Blotting Detection System Kit (Cytiva, UK) was used for developing. The bands were quantified using Image J software (Ver.1.53k). Protein extracted from Hela cells was used as a loading control.

### 4.6. Evaluation of DC Maturation and Phagocytosis by Flow Cytometry

PBMNC were isolated from peripheral blood obtained from healthy donors by density gradient centrifugation. Written informed consent was obtained for the use of blood samples from heathy donors. This study was approved by the Ethics Committee of Keio University Faculty of Pharmacy (190613-10). CD14^+^ cells selected by MACS separation system (Miltenyi Biotec) were cultured in AIM-V medium (Thermofisher Scientific, Walsam, MA, USA) supplemented with 10% AB human serum, 100 ng/mL of IL-4 (R&D Systems), and 100 ng/mL of Granulocyte Macrophage colony-stimulating Factor (GM-CSF) (R&D Systems) for 6 days. 

Those immature DCs were labelled with DiD (Invitrogen, Carlsbad, CA, USA) at 37 °C for 20 min and washed. Then, drug-treated myeloma cells were added to DC at 1:1 ratio. Maturation of DCs were evaluated by staining these DCs with anti CD86-FITC monoclonal antibody (clone B-T7; GEN-PROBE, San Diego, CA, USA), anti CD83-BV421 monoclonal antibody (clone HB15e; Biolegend, San Diego, CA, USA) or anti HLA-DR-PE monoclonal antibody (clone B-F1; Diaclone, Besanҫon, France) and analyzed by flow cytometer.

To detect phagocytosis of DCs, monocyte-derived DCs were labeled with DiD (Invitrogen) and co-cultured with GFP-expressing MUM24 treated with each drug for 48 h and percentage of DiD^+^ GFP^+^ cells was detected as an indicator of phagocytosis by DCs.

### 4.7. Carboxyfluorescein Diacetate Succinimidyl Ester (CFSE) Assay

CD14^+^ cells and CD14^−^ cells were separated from healthy donors as described above by MACS beads (Miltenyi Biotec). DCs were induced from CD14^+^ cells. CD14^−^ cells were labelled with CFSE (Thermofisher Scientific) and co-cultured with DCs pulsed with MUM24 cells, which were pretreated with each drug at IC50 for 5 days, and analyzed by flowcytometry. 

### 4.8. Microarray Analysis

MUM24 cells and KMS34 cells were treated with DMSO (0.5%) or CFZ (5 nM) for 6 h and total RNA was extracted using Isogen (Nippon Gene Co. Ltd., Tokyo, Japan) according to the manufacturer’s instruction. Clustering of differential genes from DNA microarray analysis was performed (Agilent Array, SurePrint G3 Human Gene Expression 8 × 60 K v3) using total RNA of MUM24 and KMS34 after treatment with carfilzomib, or DMSO (ArrayExpress accession E-MTAB-12797).

### 4.9. RT-PCR

Gene expression of *HSPA6* and *HMOX1* was detected by the standard reverse transcription polymerase chain reaction (RT–PCR). Total RNA was extracted from myeloma cell lines before and after treatment with bortezomib or carfilzomib using Isogen (Nippon Gene Co. Ltd., Tokyo, Japan). Complementary DNA was generated from 1 µg of the total RNA by ReverTra Ace qPCR RT Master Mix with gDNA Remover (Toyobo, Osaka, Japan) according to the manufacturer’s instructions. Then, PCR with *HSPA6* primers (forward, 5′-tggacaaggcccagattcat-3′ and reverse, 5′-atcctctccacctcctcctt-3′), *HMOX1* primers (forward, 5′-gctcaaaaagattgcccagaa-3′ and reverse, 5′-tcacatggcataaagccctaca-3′) or *beta-actin* primers (forward, 5′-atctggcaccacaccttctacaatgagctgcg-3′ and reverse, 5′-cgtcatactcctgcttgctgatccacactctgc-3′) was conducted using ExTaq polymerase (Takara Bio, Shiga, Japan). 

### 4.10. Analysis of the Overall Survival of Myeloma Patients According to Gene Expressions

Gene expression data and clinical data of myeloma patients treated with bortezomib (GSE9782) [18] was analyzed using SPSS ver.4 software. The patients were divided into high (red line) or low (blue line) quantile expressions of each gene (*HMOX1*, and *HSPA6*). Overall survival rate was compared using Kaplan–Meier survival plots.

### 4.11. Statistics

Results are presented as mean ± standard error. Two groups were compared using Student’s *t*-test. More than three groups were analyzed using ANOVA followed by Tukey’s test. Differences were considered significant when *p*-values were less than 0.05.

## 5. Patents

Bone marrow samples were collected from MM patients treated at Tokyo Saiseikai Central Hospital. Written informed consents were obtained from all patients. The use of these clinical samples was approved by ethical committee of Tokyo Saiseikai Central Hospital (No. 28-66) and the Faculty of Pharmacy, Keio University (No. 190605-3).

## Data Availability

The gene expression data and clinical data of myeloma patients presented in this study are openly available in gene expression omnibus, a public functional genomics data repository (https://www.ncbi.nlm.nih.gov/geo/query/acc.cgi?acc=GSE9782). The data of DNA microarray analysis using myeloma cell lines in this study are available on request from the corresponding author, since the data is under submission to public database.

## Supplementary Materials

The following supporting information can be downloaded at: https://www.mdpi.com/article/10.3390/ph16101367/s1, Figure S1; Direct effects of anti-myeloma drugs on human T cells. Figure S2; UPR signaling induced by PIs was suppressed by addition of the inhibitors in myeloma cells.
