# Supplementary material for: Immunomodulatory Effect of Proteasome Inhibitors via the Induction of Immunogenic Cell Death in Myeloma Cells
Source: Pharmaceuticals (Basel). 2023 Sep 27;16(10):1367. doi: 10.3390/ph16101367 (PMC10609901; doi:10.3390/ph16101367)
Supplement: Supplementary file 1 [file pharmaceuticals-16-01367-s001.zip › pharmaceuticals-2543709-supplementary.pdf]

pharmaceuticals

Supplementary Materials: Immunomodulatory Effect of Proteasome Inhibitors via the Induction of Immunogenic Cell Death in Myeloma Cells

Maiko Matsushita [1,*], Sho Kashiwazaki [1], Satoshi Kamiko [1], Michio Kobori [1], Makoto Osada [2], Hisako Kunieda [2], Maki Hirao [3], Daiju Ichikawa [1] and Yutaka Hattori [1,2]

1 Division of Clinical Physiology and Therapeutics, Faculty of Pharmacy, Keio University, Tokyo 105-8512, Japan
2 Department of Hematology, Tokyo Saiseikai Central Hospital, Tokyo 108-0073, Japan; makoosd.0221@gmail.com (M.O.)
3 Department of Health Science, Faculty of Sports and Health Science, Daito Bunka University, Saitama 355-8501, Japan
* Correspondence: matsushita-mk@pha.keio.ac.jp; Tel.: +81-3-5400-2632; Fax: +81-3-5400-2672

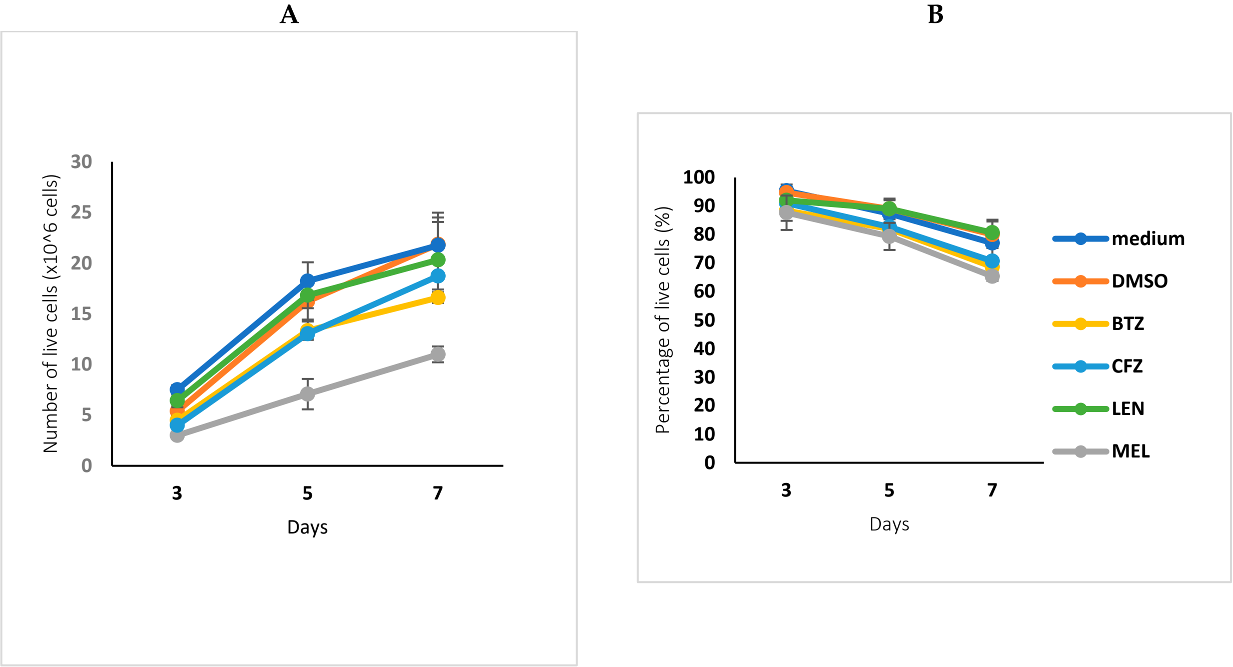

Figure S1. Direct effects of anti-myeloma drugs on human T cells. Human T cell line, Jurkat, was treated with anti-myeloma drugs at concentration of IC50 which was decided against MUM24 (MEL; 2 mM, PAN; 6 nM, CFZ; 4 nM, BTZ; 3 nM, LEN; 3.5 mM) and stained with trypan-blue. Total number (A) and percentage of live cells (B) counted 3 days, 5 days, and 7 days after addition of each drug are indicated. Data represent the mean and standard error of mean (s.e.m.) of three independent experiments. *p<0.05.

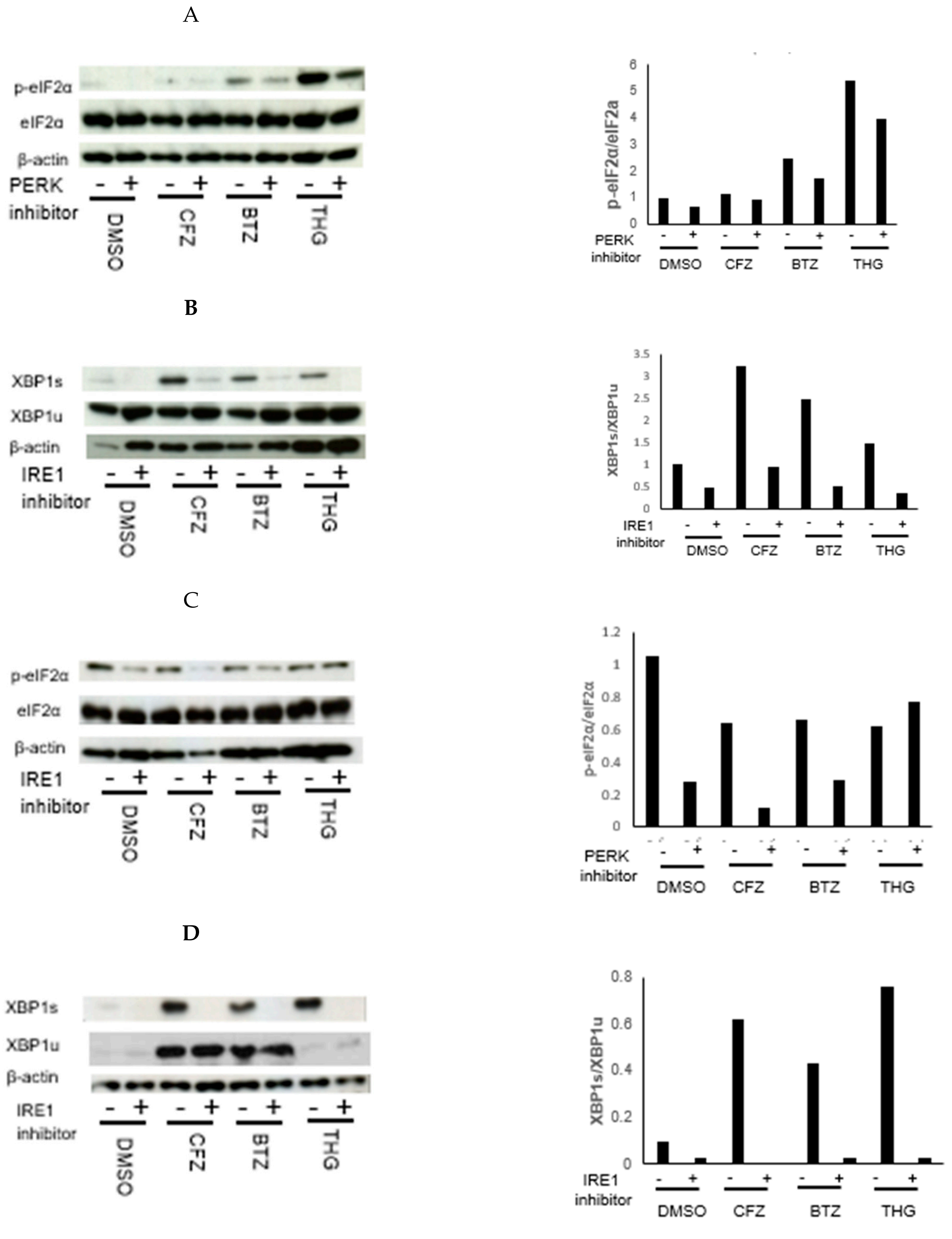

Figure S2 . UPR signaling induced by PIs was suppressed by addition of the inhibitors in myeloma cells. MUM24 cells (A,C) or KMS34 cells (B,D) were treated with PERK inhibitor (GSK2606414) or IRE1a inhibitor (STF083010) one hour before addition of proteasome inhibitors, 50 nM of BTZ, 30 nM of CFZ, and 2 μM of thapshigargin (THG) as a positive control. Phosphorylation of eIF2a (A) or splicing of XBP1 was detected using Western blot (left panel). The band quantification is also indicated (right panel). p-eIF2α ;phosphorylated eIF2α, XBP1u ;unspliced XBP1, XBP1s ;spliced XBP1.
